# MEST C Score and Treatment Response in IgA Nephropathy in a Tertiary Care Hospital: A Descriptive Cross-sectional Study

**DOI:** 10.31729/jnma.8707

**Published:** 2024-08-31

**Authors:** Sushma Thapa, Mahesh Raj Sigdel

**Affiliations:** 1 Department of Internal Medicine,Tribhuvan University Teaching Hospital, Maharajgunj, Kathmandu, Nepal

**Keywords:** *glomerulonephritis*, *IgA nephropathy*, *Oxford classification*, *renal biopsy*

## Abstract

**Introduction:**

IgA nephropathy is the leading cause of primary glomerulonephritis worldwide. The Oxford classification can predict IgA nephropathy prognosis through renal biopsy however its applicability to the Nepalese population remains unexplored. This study aimed to evaluate the MEST-C score and treatment response in patients with IgA nephropathy.

**Methods:**

This descriptive cross-sectional study was conducted at a tertiary care center from November 2021 to November 2022. Ethical approval was obtained from the Institutional Review Committee of the same institute [IRC-193(6-11)t2078/079]. Total population sampling was done. Fifty-two consenting patients aged 16 or older with confirmed IgA nephropathy were included, excluding those with liver disease or expected survival of less than six months. The study assessed the MEST-C score, demographic factors, and clinical parameters.

**Results:**

Among 52 patients with segmental glomerulosclerosis (S1), 11 (24.44%) achieved complete remission, 30 (66.67%) partial remission, and 5 (11.11%) progressed to end-stage renal disease. In those with tubular atrophy/interstitial fibrosis (T1), 1 (5.88%) achieved complete remission, 13 (76.47%) partial remission, and 4 (23.53%) progressed to end-stage renal disease. For glomerular crescents (C1), 9 (47.37%) achieved complete remission, 9 (47.37%) partial remission, and 1 (5.26%) progressed to end-stage renal disease. IFTA% of 0-25% had complete remission in 15 (46.88%). Among the two patients with IFTA% >50%, one (50%) developed end-stage renal disease and the other achieved partial remission.

**Conclusions:**

The S1 and T1/2 components of the MEST-C score had higher rates of partial remission and progression to end-stage renal disease, while other indices showed mixed results. The risk of failing to achieve complete increased with an IFTA of more than 25%.

## INTRODUCTION

IgA nephropathy (IgAN) is the most common primary glomerulonephritis in the world affecting about 200000-350000 people annually.^1,2^ IgAN was first recognized by Jean Berger (1968) after introduction of immunofluorescence techniques for renal biopsy.^2^ IgAN is likely not a single entity but rather a common response to various injurious mechanisms.

In 2009, Oxford classification, based on pathological characteristics in renal biopsies, was introduced to improve risk prediction for disease progression. Mesangial hypercellularity (M), endocapillary proliferation (E), segmental glomerulosclerosis (S), tubular atrophy/interstitial fibrosis (T) were identified as significant variables independent of clinical features.^3^ In 2017, presence of glomerular crescents (C) was added in revised Oxford classification.^4^ The percentage of the cortical area is involved by tubular atrophy /interstitial fibrosis is quantitated as Interstitial Fibrosis and Tubular Atrophy (IFTA)%. Despite its widespread use, its applicability to the Nepalese population remains unexplored.

The objective of this study was to study the MEST-C score with treatment response in patients with biopsy-proven IgA Nephropathy.

## METHODS

In this descriptive cross sectional study, a semistructured questionnaire was used to collect data from with histologically confirmed IgAN over a 12-month period at Tribhuvan University Teaching Hospital (TUTH), a tertiary care center in Nepal. After approval of the Institutional Review Committee [IRC-193(6-11)t2078/079], data were collected by the primary investigator following written informed consent. Patients aged more than 16 years were included while those with liver disease or expected survival less than 6 months due to comorbidity were excluded. Total population sampling was done.

All patients with recent biopsy proven IgAN were included during index hospitalisation or on first OPD visit with kidney biopsy reports. The baseline data and data every 4 weeks during follow-up for 6 months were collected. Treating physicians were responsible for the management and documentation. The study variables included socio-demographic variables, clinical parameters, lab parameters [complete blood count, urine red blood cell, 24-hour urine protein test, serum creatinine, serum albumin, and titres, histopathological data [light microscopy (LM), immunofluorescence study, MEST-C score, IFTA%], treatment variables (supportive treatment, immunosuppressant, renal replacement therapy), and treatment response variables (complete remission, partial remission, ESRD). Based on pathological characteristics in renal biopsies, Oxford classification identified Mesangial hypercellularity (M), endocapillary proliferation (E), segmental glomerulosclerosis (S), tubular atrophy/interstitial fibrosis (T) as significant variables independent of clinical features.^3^ Complete remission is defined as decreases in proteinuria to < 0.20 g/day with stable or improved GFR. Partial remission, where proteinuria reduces to <3.5g/day and reduction in proteinuria by 50% or more and stable or improvement in GFR and End Stage Renal Disease (ESRD), marked by an eGFR decrease to <15 ml/min.^5^

Descriptive analysis was conducted to summarize the data characteristics, including measures of central tendency and variability. Data analysis was done using IBM SPSS.

## RESULTS

A total of 52 patients were included in the study. The mean age was 34.42±11.99 years, from 17 years to 69 yrs old. Female-to-male ratio was 1.26:1 [29 females (55.77%); 23 males (44.23%)].

Out of 52 patients, 9 (17.31%) were current smokers, and 6 (11.54%) were occasional alcohol users. Hypertension was the most common finding, present in 48 patients (92.31%). Edema was observed in 47 (90.38%), and macro hematuria was seen in 5 (9.62%) patients. The lab parameters at the time of renal biopsy for 52 subjects show varied results. Haemoglobin levels averaged 12.4 ± 1.44 gm%, white blood cell count averaged 10.4 ± 7.8 x1000/cc, Platelet count averaged 2.03 ± 0.89 x100000/cc, 24-hour urine protein levels averaged 3.28 ± 1.52 gm/24 hrs, serum creatinine levels averaged 2.25 ± 1.65 mg/dl, eGFR averaged 50.31 ± 31.36 ml/min/1.73m^2^, and serum albumin levels averaged 3.16 ± 0.63 gm/dl.

Renal biopsy findings among the 52 patients revealed that the average number of glomeruli obtained in the light microscopy (LM) specimen was 14.27 ± 4.69 and 8 ± 2.00 in Immunofluorescence (IF). The findings according to the Oxford classification system (MEST-C) is shown in Table 1.

**Table 1:** Biopsy findings of IgAN patients categorised by MEST-C score (n = 52).

Biopsy Finding			n (%)
Mesangial hypercellularity	M	0	22 (42.31)
		1	30 (57.69)
Endocapillary hypercellularity	E	0	27 (51.92)
		1	25 (48.08)
Segmental glomerular sclerosis	S	0	6 (11.54)
		1	46 (88.46)
Tubular atrophy / interstitial fibrosis	T	0	18 (34.62)
		1	32 (61.54)
		2	2 (3.85)
Cellular crescents	C	0	18 (34.62)
		1	30 (57.69)
		2	4 (7.69)

Regarding treatment provided among the 52 patients, 45 (86.54%) received both immunosuppressive (IS) treatment and supportive treatment, while 7 (13.46%) received only supportive treatment. Among the 45 patients receiving IS treatment, 32 (71.11%) received only steroids while 8 (17.78%) received Mycophenolate mofetil, and 5 (11.11%) received Cyclophosphamide besides steroids. Amongst Treatment outcomes of our 52 study participants, 16 (30.77%) achieved complete remission, 31 (59.62%) achieved partial remission, and 5 (9.62%) progressed to ESRD. Among 45 patients, 24.44% achieved complete remission, 66.67% achieved partial remission, and 8.88% progressed to end-stage renal disease (Table 2).

**Table 2:** Clinical outcomes with Steroids with/without other immunosuppressants (n = 45).

Treatment	Outcome			
	CR^†^ n(%)	PR^*^ n(%)	ESRD^§^ n(%)	Total n (%)
Steroid only	10 (22.22)	20 (44.44)	2 (4.44)	32 (71.10)
Steroids + other immuno suppressive	1 (2.22)	10 (22.22)	2 (4.44)	13 (28.88)
Total	11 (24.44)	30 (66.67)	4 (8.88)	45 (100)

† CR = Complete remission

* PR = Partial remission

§ ESRD = End-stage renal disease

Lab parameters were assessed at baseline and after six months of follow-up. Notably, there was a reduction in the mean 24-hour urine protein levels, indicating an improvement in proteinuria. Additionally, both the estimated glomerular filtration rate (eGFR) and serum albumin levels showed increases, reflecting enhanced kidney function and improved nutritional status (Table 3).

**Table 3:** Lab parameters at six months with baseline values (n = 52).

Lab Parameters	Baseline	At the end of 6 months
	Mean ± SD	Mean ± SD
24 hour urine protein gm/day	3.28 ± 1.52	0.83 ± 0.57
Serum Creatinine mg/dl	2.25 ± 1.65	2.14 ± 2.31
eGFR(ml/minutes/1.73m^2^)	50.31 ± 31.36	58.81 ± 31.63
Serum Albumin gm/dl	3.16 ± 0.63	3.875 ± 0.36

In our descriptive analysis of treatment outcomes among 52 subjects, we evaluated clinical responses based on baseline levels of 24-hour urine protein (UTP) and serum creatinine. For 24-hour urine protein, mean values were 2.313 ± 0.696 g/24 hours for complete responders (CR), 3.721 ± 1.573 g/24 hours for partial responders (PR), and 3.652 ± 1.889 g/24 hours for those progressing to end-stage renal disease (ESRD). Correspondingly, mean serum creatinine levels were 1.047 ± 0.367 mg/dl for CR, 2.427 ± 1.466 mg/dl for PR, and 4.992 ± 1.674 mg/dl for ESRD.

Patients with higher grades in MEST-C indices generally showed mixed results in remission rates and progression to ESRD. Notably, segmental glomerulosclerosis (S1) and tubular atrophy/interstitial fibrosis (T1 and T2) were associated with poorer outcomes, while other indices showed varied trends. CR was achieved in almost 50% patients with T0, only 1 (1.92%) out of 18 achieved CR with T1 while no patient in T2 had CR (Table 4).

**Table 4:** Treatment outcomes and MEST G scores (n = 52).

	Sub-group	CR^†^	PR^*^	ESRD^§^
M	0	6 (11.54)	13 (25)	3 (15.79)
	1	10 (19.23)	18 (34.62)	2 (6.90)
E	0.0	6 (11.54)	17 (32.69)	4 (7.69)
	1.0	10 (19.23)	14 (26.92)	1 (3.85)
S	0.0	5 (9.62)	1 (1.92)	-
	1.0	11 (21.15)	30 (57.69)	5 (9.62)
T	0.0	15 (28.85)	17 (32.69)	-
	1.0	1 (1.92)	13 (25)	4 (7.69)
	2.0	-	1 (1.92)	1 (1.92)
C	0.0	7 (13.46)	19 (36.54)	4 (7.69)
	1.0	9 (17.31)	9 (17.31)	0 (1.92)
	2.0	-	3 (5.77)	1 (1.92)

† CR = Complete remission

* PR = Partial remission

§ ESRD = End-stage renal disease

Almost 50% of patients with IFTA% of 0-25% had a CR, while those with IFTA% of 25-50%, 13 (72.22%) had PR and only 1 (5.56%) had CR. Among the two patients with IFTA% >50%, one (50%) developed ESRD, and the other (50%) achieved partial remission.

**Table 5:** Relationship between IFTA% and clinical outcomes.

IFTA%^‡^	CR^†^	PR^*^	ESRD^§^
0-25% (n = 32)	15 (46.88)	17 (53.12)	0 (0.00)
25-50% (n = 18)	1 (5.56)	13 (72.22)	4 (22.22)
≥50% (n = 2)	-	1 (50)	1 (50)
Total	16	31	5

† CR = Complete remission

* PR = Partial remission

§ ESRD = End-stage renal disease

‡ IFTA% = Interstitial Fibrosis and T

## DISCUSSION

We could include 52 cases of IgAN in a native kidney from our single institute in a period of 12 months. The presence of IgAN amongst kidney biopsies ranges from 8.1% to 16.5% (Mittal et al.,^6^ Khairwa,^7^) in India and 2.9% to 36.6% (Garyal et al.,^8^ Subedi et al.,^9^ Manandhar et al.,^10^ Maskey et al.,^11^) in Nepal.

The mean age of the subjects in our study was 34.42±11.99 years. A similar finding was obtained by Subedi et al.^9^ in Nepal (mean age 35.37 years). The mean age of the patients varied more in studies from India, (27.2±16.7 to 48.6±21.3 years) in different studies.^6,7^ Most patients were in the age group 16-29 years (20, 38.5%) and 30-39 years (19, 36.5%). Our sample had 29 female and 23 male, female to male ratio being 1.26:1. Most studies^6,7^ have shown male preponderance for IgAN while others^12,13^ have found no difference or female preponderance. For example, a study done by Deng et al. found the ratio of F: M as 1.4:1.^12^

Activated mesangial cells in IgAN release mediators of renal injury that can lead to damage in the podocytes and proximal tubule epithelial cells, increased glomerular permeability, and scarring in the glomerular and interstitial compartments, resulting in hypertension, proteinuria, hematuria, and reduced renal clearance.^14,15^ We observed that hypertension was the most common finding (92.31%) followed by edema (90.38%), similar to the studies done by Mittal et al (78.8%)^6^ Gowrishankar et al (60.45%)^16^ and Chako et al (58%).^17^

Persistent proteinuria has been identified to be one of the most important clinical risk factors for poor prognosis, as well as hypertension and renal insufficiency, while diffuse mesangial proliferation, segmental sclerosis, crescents (C), interstitial fibrosis, and tubular atrophy (T) are considered to be pathological risk factors.^18,19^ The mean 24-hour urine protein at presentation in our study was 3.28 ± 1.52 gm/day. Proteinuria >3.5gm/day was found in 20 (38.5%) subjects. Chen et al.^20^ showed a mean 24-hour urine protein of 3.51 gm/day, Neelakantappa et al.^21^ reported that 69% IgAN had nephrotic range proteinuria while Mittal et al. showed 23% of patients had nephrotic range proteinuria.^6^ These along with our findings suggest that nephrotic range proteinuria is fairly common in IgAN.

In our study, 16 (30.76%) achieved complete remission, 31 (59.62%) achieved partial remission, and 5 (9.62%) developed ESRD. In the study by Chako et al.ESRD was present in 17% patients.^17^ In the study by Rauen et al.,ESRD was present in 29.40% patients.^22^ Taking CR and PR together, the response was seen in 90.38%. Short-term follow-up studies have reported a correlation between early response in proteinuria and eGFR with long-term renal outcome.^5,18,22,26^ However, this does not necessarily mean that early response translates into long-term benefits in terms of renal survival. This is the fallacy of short-term follow-up studies and is a limitation when predicting long-term outcomes. The severity of tubular atrophy/interstitial fibrosis (T), segmental glomerulosclerosis (S), and cellular/fibrocellular crescents (C) indicate squeal of inflammation and ongoing kidney damage and can predict worse long-term outcomes, including progression to ESRD.^23^ Without appropriate treatment, the continued presence of these features can lead to further kidney damage and a decline in renal function.

In various studies, the association between the MEST-C score and treatment response has been found to vary. A study done by Lv et al.^24^ reported a highly significant relationship between the MEST-C score and ESRD in the S1 subgroup and a significant relationship in the T1/2 subgroup, while no association was found with ESRD in the E subgroups. Similarly, in studies by Alamartine et al.,^25^ and Haaskjold et al.^26^, the MEST-C score of S1 and T1/2 was found to be significant in relation to treatment response. Furthermore, Haaskjold et al.26 found that the MEST-C score significantly affected the outcome. Schimpf et al.^27^ found that the MEST-C score M1 was associated with worse outcomes, and ESRD was more prevalent in the T1/T2 group, while C1/C2 was associated with worse outcomes only when steroids were not used. Itami et al.^28^ reported that the MEST-C T1/T2 score had no treatment response, even when steroids were used. In Coppo et al.,^29^ T1/T2, M1, and S1 of the MEST-C score were found to be significant in relation to treatment response if steroids were not used.

Overall, the association of the MEST-C score with treatment response is not consistent across different studies; however, Scores of S1 and T1/T2 portend poorer outcomes. In the study by Rauen et al.^22^, 17% of patients who received IS treatment and 5% of patients who received supportive treatment had complete remission. The study by Itami et al.^28^ found that the use of a steroid was useful, with a 75.5% 20-year survival rate compared to 61% for those not receiving the steroid. The study by Coppo et al.^18^ and Lv et al.^30^ found that a combination of a steroid with an ACEi was better than an ACEi alone. Judicious use of immunosuppression in carefully selected patients should be beneficial.

Our study found that almost 50% of patients with IgAN with IFTA <25% achieved CR. More than 70% of patients with IFTA 25-50% had PR. Among the two patients with IFTA% ≥50%, one developed ESRD, and the other achieved partial remission. The IFTA percentage can be a prognostic indicator and a predictor of the clinical outcome. ^31,32^ Zhu et al.^31^ and Silva et al.^32^ both found that IFTA is the most significant prognostic factor for the outcome of patients with IgAN. Both studies suggest that patients with a higher IFTA are more likely to have a poorer outcome and progress to (ESRD) compared to those with a lower IFTA.

The limitation of the studies includes small sample size, patients from single tertiary care centre. A longer followup period could have been more effective in predicting long term outcome, recurrence and treatment effectiveness.

## CONCLUSIONS

IgA nephropathy is a very important cause of glomerulonephritis in our setup. The mean age was 34.42 years with a female-to-male ratio of 1.26:1. Hypertension, hematuria and edema were the three most important presentations. The mean creatinine, eGFR, serum albumin, and UTP at baseline were 2.25 mg/dl, 50.31 ml/min/1.73m^2^, 2.16gm/dl, and 3.28 gm/24hrs. Most of the patients had either complete or partial remission. Out of different components of the MEST-C score S1 and T1/2 components had higher rates of partial remission and progression to ESRD.

The risk of failure to achieve CR increased with an IFTA of more than 25%. Further research with larger sample sizes and longer follow-up periods is recommended to better understand the application of MEST C score in the managment of IgA nephropathy.

## References

[1] McGrogan A, Franssen CF, de Vries CS (2011). The incidence of primary glomerulonephritis worldwide: a systematic review of the literature.. Nephrol Dial Transplant..

[2] Schena FP, Nistor I (2018). Epidemiology of IgA nephropathy: A global perspective.. Semin Nephrol.

[3] Cattran DC, Coppo R, Cook HT (2009). The Oxford classification of IgA nephropathy: rationale, clinicopathological correlations, and classification.. Kidney Int..

[4] Trimarchi H, Barratt J, Cattran DC (2017). Oxford classification of IgA nephropathy 2016: an update from the IgA nephropathy classification working group.. Kidney Int..

[5] Marques F, Reis J, Godinho I, Pereira M, Fernandes P, Jorge S (2022). Impact of Early Proteinuria Reduction in Glomerular Disease and Decline of Kidney Function: A Retrospective Cohort.. J Clin Med Res [Internet]..

[6] Mittal N, Joshi K, Rane S (2012). Primary IgA nephropathy in north India: is it different?. Postgrad Med J..

[7] Khairwa A (2021). Indian scenario of IgA nephropathy: a systematic review and meta-analysis.. Afri Health Sci..

[8] Garyal, Kafle RK (2008). Hisopathological spectrum of glomerular disease in Nepal: a seven-year retrospective study.. Nepal Med Coll J..

[9] Subedi M, Bartaula B, Pant AR (2018). Pattern of glomerular disease and clinicopathological correlation: A single-center study from Eastern Nepal.. Saudi J Kidney Dis Transpl..

[10] Manandhar DN, Chhetri PK, Poudel P (2016). Spectrum of glomerular diseases in native kidneys in patients attending Nepal Medical College Teaching Hospital.. J Adv Intern Med..

[11] Maskey A, Lamsal L (2019). Pattern of glomerular diseases in biopsy proven native kidney in Western Nepal.. J Adv Intern Med..

[12] Deng W, Tan X, Zhou Q (2018). Gender-related differences in clinicopathological characteristics and renal outcomes of Chinese patients with IgA nephropathy.. BMC nephrology..

[13] Shen P, Ding X, Ten J (2006). Clinicopathological characteristics and outcome of adult patients with hematuria and/or proteinuria found during routine examination.. Nephron Clinical Practice..

[14] Wyatt RJ, Julian BA (2013). IgA Nephropathy.. N Engl J Med..

[15] Pattrapornpisut P, Avila-Casado C, Reich HN (2021). IgA nephropathy: core curriculum 2021.. Am J Kidney Dis.

[16] Gowrishankar S, Gupta Y, Vankalakunti M (2019). Correlation of Oxford MEST-C Scores With Clinical Variables for IgA Nephropathy in South India.. Kidney International Reports..

[17] Chacko B (2011). IgA nephropathy in India: what we do know.. Renal Failure..

[18] Coppo R, Lofaro D, Camilla RR (2017). Risk factors for progression in children and young adults with IgA nephropathy: an analysis of 261 cases from the VALIGA European cohort.. Pediatr Nephrol.

[19] Barbour S, Reich H (2018). An update on predicting renal progression in IgA nephropathy.. Curr Opin Nephrol Hypertens.

[20] Chen Y, Yang A, Hou Y (2022). Comparison between outcomes of IgA nephropathy with nephrotic-range proteinuria and nephrotic syndrome: do podocytes play a role?.. Renal Failure..

[21] Neelakantappa K, Gallo GR, Baldwin DS (1988). Proteinuria in IgA nephropathy.. Kidney international..

[22] Rauen T, Eitner F, Fitzner C (2015). Intensive supportive care plus immunosuppression in IgA nephropathy.. N Engl J Med..

[23] Roberts IS (2014). Pathology of IgA nephropathy.. Nat Rev Nephrol..

[24] Lv J, Shi S, Xu D (2013). Evaluation of the Oxford Classification of IgA nephropathy: a systematic review and meta-analysis.. Am J Kidney Dis..

[25] Alamartine E, Sauron C, Laurent B (2011). The use of the Oxford classification of IgA nephropathy to predict renal survival.. Clin J Am Soc Nephrol.

[26] Haaskjold YL, Bjorneklett R, Bostad L, Bostad LS, Lura NG, Knoop T (2022). Utilizing the MEST score for prognostic staging in IgA nephropathy.. BMC Nephrol..

[27] Schimpf JI, Klein T, Fitzner C (2018). Renal outcomes of STOP-IgAN trial patients in relation to baseline histology (MEST-C scores).. BMC Nephrology..

[28] Itami S, Moriyama T, Miyabe Y (2022). A novel scoring system based on Oxford classification indicating steroid therapy use for IgA nephropathy.. Kidney international reports..

[29] Coppo R, Troyanov S, Bellur S (2014). Validation of the Oxford classification of IgA nephropathy in cohorts with different presentations and treatments.. Kidney Int..

[30] Lv J, Zhang H, Chen Y (2009). Combination therapy of prednisone and ACE inhibitor versus ACE-inhibitor therapy alone in patients with IgA nephropathy: a randomized controlled trial.. Am J Kidney Dis..

[31] Zhu X, Li H, Liu Y (2017). Tubular atrophy/interstitial fibrosis scores of Oxford classification combinded with proteinuria level at biopsy provides earlier risk prediction in lgA nephropathy.. Sci Rep..

[32] Silva C, Afonso N, Cotovio P (2012). Prognostic factors in adult patients with idiopathic IgA Nephropathy.. Revista Portuguesa de Nefrologia e Hipertensäo..

